# Collision-Free Advertisement Scheduling for IEEE 802.15.4-TSCH Networks †

**DOI:** 10.3390/s19081789

**Published:** 2019-04-14

**Authors:** Apostolos Karalis, Dimitrios Zorbas, Christos Douligeris

**Affiliations:** 1 Department of Informatics, University of Piraeus, 18534 Piraeus, Greece; cdoulig@unipi.gr; 2 Tyndall National Institute, University College Cork, T12R5CP Cork, Ireland; dimitrios.zormpas@tyndall.ie

**Keywords:** IEEE802.15.4-TSCH, network formation, mobility, IIoT

## Abstract

IEEE802.15.4-time slotted channel hopping (TSCH) is a medium access control (MAC) protocol designed to support wireless device networking, offering high reliability and low power consumption, two features that are desirable in the industrial internet of things (IIoT). The formation of an IEEE802.15.4-TSCH network relies on the periodic transmissions of network advertising frames called enhanced beacons (EB). The scheduling of EB transmissions plays a crucial role both in the joining time and in the power consumption of the nodes. The existence of collisions between EB is an important factor that negatively affects the performance. In the worst case, all the neighboring EB transmissions of a node may collide, a phenomenon which we call a full collision. Most of the EB scheduling methods that have been proposed in the literature are fully or partially based on randomness in order to create the EB transmission schedule. In this paper, we initially show that the randomness can lead to a considerable probability of collisions, and, especially, of full collisions. Subsequently, we propose a novel autonomous EB scheduling method that eliminates collisions using a simple technique that does not increase the power consumption. To the best of our knowledge, our proposed method is the first non-centralized EB scheduling method that fully eliminates collisions, and this is guaranteed even if there are mobile nodes. To evaluate our method, we compare our proposal with recent and state-of-the-art non-centralized network-advertisement scheduling methods. Our evaluation does not consider only fixed topology networks, but also networks with mobile nodes, a scenario which has not been examined before. The results of our simulations demonstrate the superiority of our method in terms of joining time and energy consumption.

## 1. Introduction

The wireless applications of the industrial internet of things (IIoT) require the networking of power-constrained wireless devices under stringent reliability, availability, and security requirements [1,2]. To support these applications, even in harsh industrial environments, the IEEE802.15.4 standard [3] proposes a specialized medium access method named time slotted channel hopping (TSCH). TSCH is a deterministic protocol that combines schedule-based communication with a slow channel hopping mechanism in order to achieve ultra-low power consumption and ultra-high reliability.

The formation of an IEEE802.15.4-TSCH network relies on the periodic transmission of enhanced beacons (EB). EB are special broadcast frames sent by the network nodes in order to advertise its existence. Whether a node sends EB depends on the capabilities of the specific node and the network design. For convenience, we call *advertisers* the nodes that send EB. Initially, the network contains only the personal area network (PAN) coordinator, which is the central advertiser of the network. A *joining node*, which is a node desiring to join the network, turns its radio on and scans the available channels for EB. The reception of an EB is necessary for the initial synchronization of the node with the network. Although EB can be sent on a predefined static channel, this may cause a node to fail to join the network when, for instance, this channel is blocked in case of interference. Similarly, a joining node may not be able to join the network if the node constantly listens for EB on a randomly selected channel. Due to the use of multiple orthogonal channels and the channel-hopping mechanism, finding an EB may require a considerable amount of time. The longer the time it takes the joining node to find an EB, the higher the energy the node consumes. The capability to adjust the EB transmission rate allows the achievement of a trade-off between the joining time and the energy consumption of the advertisers [4]. Furthermore, the increase of the EB transmission rate typically requires the allocation of more communication resources, leading to a reduction in communication resources available for data transmission or for data reception.

It must be noted here that for the secure joining of a node in the network, it is not enough to receive an EB, that is to synchronize with the network, but also a security protocol is required to run afterwards for authentication, authorization and security parameter distribution. In this paper, we focus only on the synchronization part of the joining procedure, which is affected by the EB scheduling.

Although EB scheduling is of major importance for the formation of IEEE802.15.4-TSCH networks, the standard [3] does not define any EB scheduling method, but it assumes that the EB rate is configured by a higher layer as appropriate to the density of the nodes, the desired time for network formation, and the energy devoted to network formation. As a consequence, several scheduling methods [5,6,7,8,9,10,11,12,13,14,15] have been proposed in the literature.

A critical issue for the decline of the performance of an EB scheduling method is the existence of collisions during the transmissions of EB. Apparently, the collisions have a significant negative impact on the probability of successful EB reception, resulting in longer joining times and in increased power consumption. In the worst case, all the neighboring EB transmissions of a joining node collide, a phenomenon which we call *full collision*.

In this paper, we firstly provide a mathematical analysis of the collision probability when the EB scheduling is based on randomness, a fact that holds true in most of the methods proposed in the literature so far. Specifically, we examine the probability of a collision between EB transmissions as well as the full collision probability. The purpose of this analysis is to show that, although randomness can be a simple tool to construct the EB schedule fast and with negligible power consumption, it may lead to a high probability of collisions, and, especially, of full collisions, which may result in very long joining times. After this analysis, we propose a novel autonomous “collision-free advertisement scheduling” (CFAS) method. CFAS is characterized as autonomous since each advertiser performs EB scheduling autonomously, that is, without any negotiation with its neighbors. To the best of our knowledge, this is the first non-centralized collision-free method. With CFAS, each advertiser constructs its EB schedule based on its identifier, and the collision avoidance is ensured even if the nodes are moving.

At this point, it is worth mentioning that, despite the fact that mobility is important for many applications [16], none of the previous studies on EB scheduling has examined the case of mobile nodes. Since mobility causes changes in the topology of a network, the EB schedule should be adjusted in these changes in order to minimize collisions. CFAS guarantees that, when an advertiser transmits an EB, no other node in the network transmits in the same channel simultaneously and, thus, the EB schedule does not need to change because of any likely topology changes.

However, the use of non-shared communication resources for EB implies that a larger portion of communication resources should be allocated for EB and, by extension, a smaller portion of those would remain available for data. To tackle this problem, we integrate our previously proposed “advertisement timeslot partitioning” (ATP) [17] technique into CFAS. ATP is an optimization technique for EB transmissions, which allows CFAS to significantly reduce the amount of communication resources needed for EB, without compromising the resulting performance.

To evaluate the performance of our proposed method, we compare it with the two most recently proposed non-centralized advertisement scheduling methods that are purely intended for IEEE802.15.4-TSCH. These are the “enhanced coordinated vertical filling” (ECV) and the “enhanced coordinated horizontal filling” (ECH) [5]. In addition, we make a comparison with the well-known minimal 6TiSCH (IPv6 over IEEE802.15.4-TSCH) configuration [4]. It should be noted that we take into account both the cases of a fixed and of a mobile joining node. As far we know, this is the first paper that examines the joining procedure of IEEE802.14.5-TSCH from the perspective of a mobile node. Our simulations demonstrate the capability of our method to achieve much shorter average joining times with the same or lower power consumption on advertisers. The cost of the shorter average joining time is translated to a higher portion of communication resources allocated for EB, but as we previously said, this overhead is significantly reduced through ATP.

In summary, the contributions of our paper are the following:

- We provide a mathematical analysis of the collisions between EB when the scheduling is based on randomness, in order to show that randomness may lead to a considerably high probability of collision, and, especially, of a full collision, which may result to very long joining times.
- We propose a novel autonomous EB scheduling method that fully eliminates collisions through a simple mechanism which does not increase the power consumption of the nodes. Compared to other non-centralized methods proposed in the literature, our method is the first that optimizes the joining time through the complete elimination of collisions, while the collision avoidance is guaranteed even if there are mobile nodes. Additionally, in order to minimize the communication resources required by our method, we utilize a recently proposed optimization technique, namely ATP.
- We evaluate through simulations the performance of our method compared to ECV and ECH, which are the two most recently proposed non-centralized advertisement scheduling methods that are purely intended for IEEE802.15.4-TSCH. Moreover, we make a comparison with the well-known 6TiSCH minimal configuration. Our evaluation is not limited to the case of a fixed joining node, but we also take into account the case of a mobile joining node. The results of our simulations show the superiority of our method in all the examined cases.

The remainder of the paper is structured as follows. In Section 2 we give a brief overview of the IEEE802.15.4-TSCH operation, and in Section 3 we present the related work on EB scheduling. Afterwards, in Section 4 we deal with the aforementioned collision analysis. In Section 5 we present CFAS and how it can be enhanced with ATP. Section 6 evaluates the performance of our proposed method and presents the comparison results. Finally, Section 7 concludes this paper and outlines ideas for future work.

## 2. Overview of IEEE802.15.4-TSCH

IEEE802.15.4-TSCH is a medium access control (MAC) protocol where the nodes communicate through a schedule built on a slotframe structure; that is, a collection of equal-length timeslots that repeats cyclically. According to the schedule, each node knows in which timeslots it must be active to receive or send a frame, while in the others it turns its radio off to save energy. For each timeslot in which a node is active, the schedule defines a pseudo-channel called *channel offset*, which is utilized for the calculation of the physical channel that the node will use. The channel calculation is performed via the following equation [1]:

(1) PhysicalChannel=F{(ASN+ChannelOffset)%C},

where ASN is the absolute slot number, which denotes the total number of timeslots that have elapsed since the start of the network, *C* is the number of available channels (e.g., 16 when the 2.4 GHz frequency band is used and all the band’s channels are available), ChannelOffset takes integer values between 0 and C−1, and *F* is a bijective function mapping an integer between 0 and C−1 into a physical channel. Equation (1) performs a slow channel hopping in order to minimize the negative effects of noise and interference, aiming to provide high reliability. Considering that the number of timeslots in the slotframe is not a multiple of C, Equation (1) returns a different channel for the same pair of timeslot and channel offset at each slotframe cycle. When the slotframe length and C are relatively prime, each pair of timeslot and channel offset rotates over the available channels as the slotframe repeats.

The communication schedule is depicted as a two-dimensional matrix, where the rows represent the channel offsets and the columns represent the timeslots. Each cell of the matrix is a discrete communication resource, which can be dedicated or shared. A dedicated cell is reserved for the transmissions of a single node, while in a shared cell multiple nodes can transmit, and, thus, collisions may arise. The cell allocation is performed based on the needs of the applications running on the network, that is, on the needs of data transmissions as well as on the transmission needs of the control messages (e.g., EB) of TSCH and higher level protocols.

**Figure 1:**
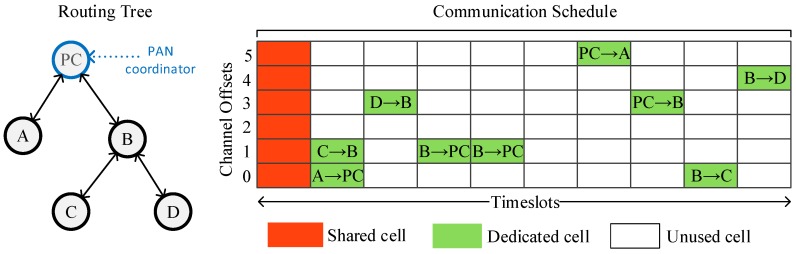
A five-node topology with a simple time slotted channel hopping (TSCH) schedule using dedicated cells for unicast transmissions and shared cells for broadcast transmissions.

An example of a schedule is shown in Figure 1. In this example, there are five nodes in the network, the cells of the first timeslot have been marked as shared and used for broadcast frames, such as EB, while unicast transmissions (e.g., data transmissions) take place in the dedicated cells.

## 3. Related Work

De Guglielmo et al. [14] conduct a performance analysis on the formation of an IEEE802.15.4-TSCH network through a simple random-based advertisement algorithm allocating only one timeslot for EB. To minimize collisions, each node transmits EB with a probability that depends on the number of neighboring advertisers transmitting with the same channel offset.

De Guglielmo et al. [11] formulate an optimization problem to calculate the optimal EB cells, defined as the cells in which each advertiser should transmit EB in order to achieve the minimum average joining time. However, as they observe, their approach may lead to a large number of collisions and may require an advertiser to transmit on multiple channels in the same timeslot. For these reasons, they propose the alternative “model-based beacon scheduling” (MBS) approach, where each advertiser transmits in only one of the optimal cells, which is randomly selected by the advertiser. The optimal cells are calculated by the PAN coordinator and are propagated to the other advertisers via EB.

Khoufi and Minet [6] propose a centralized collision-free EB scheduling algorithm called “enhanced deterministic beacon advertising” (EDBA). This is an enhanced version of the “deterministic beacon advertising” (DBA) algorithm presented in Ref. [10]. When EDBA is used, the advertisement cells (i.e., cells allocated for EB) are regularly spaced in the slotframe, the PAN coordinator transmits EB in the cell of the first timeslot having channel offset 0, while any other advertiser transmits in a cell computed by the PAN coordinator during its association to the network.

Duy et al. [13] propose a scheduling scheme allowing the dynamic adjustment of the EB rate. According to this scheme, the EB schedule is built on a multi-slotframe structure, where each slotframe is divided into two parts: the advertisement plane and the data plane. The advertisement plane is the first part of the slotframe and it consists of advertisement slots (i.e., slots used for EB). On the other hand, the slots of the data plane are used only for data transmissions. The size of the advertisement plane is selected based on the current network requirements and determines the maximum possible EB rate of an advertiser. Based on the desired EB rate, each advertiser transmits EBs in a defined number of slots, using consecutive physical channels, starting from a randomly selected channel. On top of the above scheme, the authors propose a fuzzy-logic mechanism adjusting the EB rate based on the number of advertisers contained in the network [12]. The goal is to dynamically adjust the EB rate in a way that minimizes the power consumption of both the joining nodes and the advertisers. We must note, however, that their mechanism assumes that all the nodes are visible to each other.

Kim et al. [8] propose a fast joining scheme based on channel quality, for environments with severe interference. This scheme utilizes the multi-slotframe structure proposed in [13], with the difference that the last two slots of each slotframe are used for channel quality estimation. A joining node uses only the best channel to receive an EB, while the advertisers transmit EB in the best *n* channels, where *n* is a parameter of the proposed scheme. Both the joining nodes and the advertisers periodically re-estimate the quality of the channels. Although, their mechanism seems to achieve a low average joining time in high interference environments, it requires additional power consumption by both the advertisers and the joining nodes, a problem which has not been evaluated.

Vogli et al. [5] propose four techniques to minimize the joining time. These are “random vertical filling” (RV), “enhanced coordinated vertical filling” (ECV), “random horizontal filling” (RH), and “enhanced coordinated horizontal filling” (ECH). All of them use a multi-slotframe structure, where only the first slot of each slotframe is an advertisement slot. In RV and RH the EB schedule is created randomly, while in ECV and ECH the advertisers sense the advertisement cells in order to find a free one, and, thus, to avoid collisions. It is should be noted that the authors consider that the nodes are visible to each other and they do not take into account the hidden node problem. The difference between RV and RH, as well as between ECV and ECH, is the way the advertisement cells are filled. Time-vertical and horizontal approaches are proposed. Because of their collision avoidance technique, ECV and ECH achieve much better average joining times compared to RV and RH. However, they increase the power consumption of the advertisers, and they do not fully eliminate collisions.

Vucinic et al. [9] consider the EB transmissions in the context of the minimal 6TiSCH configuration, which recommends the use of only one shared cell for bootstrapping and broadcast traffic. Their goal is to lower the contention within the shared slot by tuning the transmission probability of the different kinds of frames (e.g., EB). In this direction, they propose a practical adaptation of the Bayesian broadcast algorithm [18] to 6TiSCH networks.

Vallati et al. [7] present a work on EB scheduling under the minimal 6TiSCH configuration. Initially, the authors show that the static allocation of only one shared cell can lead to poor performance. To tackle this problem, they propose the allocation of multiple shared cells through a dynamic strategy that adapts the number of shared cells allocated by each node based on an estimation of the rate of the control messages transmitted within a neighborhood. They propose a dynamic, rather than a static, strategy in order to manage the trade-off between network formation performance and resource utilization. Their performance evaluation shows that the proposed algorithm can enhance the reliability and the efficiency of the network formation procedure.

Finally, Vera–Perez et al. [15] carry out an experimental study to find the optimal EB rate in terms of joining time and power consumption. As a result of their simulations on a specific topology, they proposed a dynamic adaptation mechanism for the EB rate, called “custom trickle timer”. Their mechanism uses a high EB rate at the first minutes of the network operation, and a very low EB rate afterwards. Compared to the static use of the same high EB rate, their mechanism achieves similar joining times, but with a much lower power consumption. However, their solution cannot be considered as a general solution since it is influenced by their assumed topology and the implications of such an assumption.

In this paper, we propose a new autonomous collision-free technique. In relation to the methods proposed in the literature, our method reuses the idea of the multi-slotframe structure [5,12,13] and the idea of an increased EB rate of the PAN coordinator when the PAN coordinator has no power limitations, which has been proposed in Ref. [5]. Moreover, it utilizes the general idea of scheduling based on node identifiers, an idea that has already been utilized in unicast transmissions between neighbors, such as in Ref. [19]. Our method combines the above ideas in a way that allows the creation of a collision-free EB scheduling with a simple way that does not increase power consumption. Compared to the non-centralized methods that have been proposed in the literature so far, our method is the only one that can speed-up the joining procedure through the full elimination of collisions. For the sake of completeness, it should also be pointed out that the idea of using the nodes’ identifiers for providing collision-free transmissions has also been utilized by the DeBras-TDMA algorithm proposed in Ref. [20]. However, this algorithm focusses on providing collision-free transmissions for DeBras messages, that is for the special frames of the algorithm that locally broadcast scheduling information, and not for the EB.

## 4. Collision Analysis

The collision probability is an important performance factor of an EB scheduling method. Generally, the higher the collision probability, the lower the probability an EB to be successfully received. Nevertheless, the majority of the methods that have been proposed so far do not use a collision avoidance mechanism, but they treat collisions as random events. However, as we will see below, this tactic can lead to a high collision probability. We assume that we have a network where there are $C\in N_{\neq 0}$ advertisement cells and each advertiser transmits EBs in a randomly selected cell. Moreover, we assume that there is a joining node with $N\in N_{\neq 0}$ neighboring advertisers. To avoid collisions between the EB transmissions, the neighboring advertisers must select different cells. Of course, this can happen only if C≥N. In this case, the number of possible ways that the neighboring advertisers can select different cells is equal to the *N*-permutations of *C*, and since all the possible ways that the cells can be selected by the neighboring advertisers is $C^{N}$, the probability of no collisions is $\frac{C!}{C^{N}\left(C-N\right)!}$. Consequently, the probability of a collision (or, equivalently, the probability that the EB schedule is not collision-free) is given by Equation (2).

(2) $$P\left(\mathrm{collision}\right)=\left\{\begin{array}{cc} 1-\frac{C!}{C^{N}\left(C-N\right)!}, & \mathrm{if}\;C\geq N\\ 1, & \mathrm{otherwise}. \end{array}\right.$$

**Figure 2:**
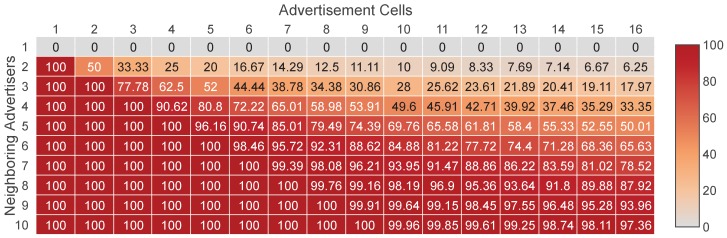
Probability of a collision (%) between the neighboring enhanced beacons (EB) transmissions of a joining node, when each advertiser transmits in a randomly selected advertisement cell.

Figure 2 shows the probability of a collision for different numbers of neighboring advertisers and advertisement cells. The range of the advertisement cells is selected based on the EB scheduling methods that have been proposed in the literature. As shown, the probability of a collision is very high in most cases. To achieve a low collision probability, the number of advertisement cells must be much greater than the number of neighboring advertisers, which, for obvious reasons, is not a generally acceptable solution.

In the worst case of EB collisions, all the neighboring EB transmissions of the joining node collide, a phenomenon which we call full collision. A full collision takes place not only when all the neighboring EB transmissions take place in a single cell, but also when they happen in multiple advertisement cells and each of these cells is used by more than one neighboring advertisers. In the latter case, the neighboring advertisers of the joining node collide in groups. Therefore, in general, the full collision probability is given by the following formula:

(3) P(FullCollision)=P(FullCollisioninasinglecell)+P(FullCollisioninmultiplecells).

Obviously, a full collision can occur only when N>1. Subsequently, we examine the cases where N>1. Initially, we calculate the probability of a full collision in a single cell. Since each neighboring advertiser uses a randomly selected cell, there are *C* ways for a full collision in a single cell. Therefore, the probability is computed as follows:

(4) $$P\left(\mathrm{Full}\;\mathrm{Collision}\;\mathrm{in}\;a\;\mathrm{single}\;\mathrm{cell}\right)=\frac{C}{C^{N}}=\frac{1}{C^{N-1}}\;\mathrm{if}\;N>1.$$

A full collision in multiple cells can occur only when C>1 and N>3 and consequently considering Equations (3) and (4) follows that:

(5) $$P\left(\mathrm{Full}\;\mathrm{Collision}\right)=\left\{\begin{array}{cc} 1 & \mathrm{if}\;N>1\;\mathrm{and}\;C=1\\ \frac{1}{C^{N-1}} & \mathrm{if}\;1<N<4\;\mathrm{and}\;C>1. \end{array}\right.$$

Then we examine the cases where C>1 and N>3. From a mathematical point of view, a full collision in multiple cells appears when the neighboring advertisers are partitioned into groups that contain at least two members and use different cells. It is clear that such a partitioning can create from two to $\min\left(\left\lfloor N/2\right\rfloor ,C\right)$ groups. In order to find the number of all the possible partitioning ways, we will utilize the two-associated Stirling numbers of the second kind [21]. A two-associated Stirling number of the second kind, which is denoted by $S_{2}\left(n,k\right)$, expresses the number of ways a set with *n* elements can be partitioned into *k* disjoint subsets of at least two elements. The two-associated Stirling numbers of the second kind are an integer sequence and an explicit formula for their calculation is the following [22]:

(6) S2n,k=∑i=0k−1ini∑j=0k−i−1jk−i−jn−ij!k−i−j!,n≥2,1≤k≤n2.

Utilizing Equation (6), we can count the possible partitions of the neighboring advertisers into groups of at least two members, through the sum $\sum_{k=2}^{\min\left(\left\lfloor N/2\right\rfloor ,C\right)}S_{2}\left(N,k\right)$. For each possible partition into k groups, there are $\frac{C!}{(C-k)!}$ ways that the groups select different cells. Consequently, the total number of cases where a full collision occurs in multiple cells is $\sum_{k=2}^{\min\left(\left\lfloor N/2\right\rfloor ,C\right)}S_{2}\left(N,k\right)\frac{C!}{(C-k)!}$ and, thus, the corresponding probability is:

(7) $$P\left(\mathrm{Full}\;\mathrm{Collision}\;\mathrm{in}\;\mathrm{multiple}\;\mathrm{cells}\right)=\frac{\sum_{k=2}^{\min\left(\left\lfloor N/2\right\rfloor ,C\right)}S_{2}\left(N,k\right)\frac{C!}{(C-k)!}}{C^{N}},\;\mathrm{if}\;N>3\;\mathrm{and}\;C>1.$$

By combining Equations (3), (4) and (7), it follows that:

(8) $$P\left(\mathrm{Full}\;\mathrm{Collision}\right)=\frac{1}{C^{N-1}}+\frac{\sum_{k=2}^{\min\left(\left\lfloor N/2\right\rfloor ,C\right)}S_{2}\left(N,k\right)\frac{C!}{(C-k)!}}{C^{N}},\;\mathrm{if}\;N>3\;\mathrm{and}\;C>1.$$

In summary, by taking into account Equations (5) and (8), as well as the fact that a full collision does not occur when N=1, Equation (9) gives the complete formula of the full collision probability:

(9) $$P\left(\mathrm{Full}\;\mathrm{Collision}\right)=\left\{\begin{array}{cc} 0 & \mathrm{if}\;N=1\\ 1 & \mathrm{if}\;N>1\;\mathrm{and}\;C=1\\ \frac{1}{C^{N-1}} & \mathrm{if}\;1<N<4\;\mathrm{and}\;C>1\\ \frac{1}{C^{N-1}}+\frac{\sum_{k=2}^{\min\left(\left\lfloor N/2\right\rfloor ,C\right)}S_{2}\left(N,k\right)\frac{C!}{(C-k)!}}{C^{N}} & \mathrm{otherwise}. \end{array}\right.$$

**Figure 3:**
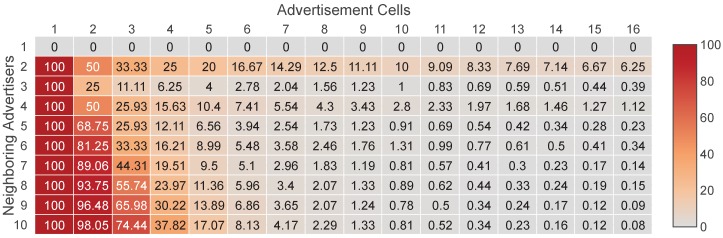
Full collision probability (%) between the neighboring EB transmissions of a joining node, when each advertiser transmits in a randomly selected advertisement cell.

In Figure 3 we present the full collision probability for *N* from one to 10 and with up to 16 advertisement cells. As we can observe, the probability is not negligible when the number of available cells is low. A typical scenario of this case appears when the available cells are limited because multiple channels have been blacklisted due to the presence of external interference [23].

## 5. Collision Free Advertisement Scheduling (CFAS)

In order to minimize the node joining time through the elimination of collisions, we propose CFAS. Our proposed method ensures the elimination of collisions via a simple mechanism that does not increase the power consumption, neither does require any negotiation between the nodes. Moreover, the collision avoidance is guaranteed even if there are mobile nodes. To the best of our knowledge, our proposed method is the first non-centralized EB scheduling method that provides collision-free EB transmissions.

According to CFAS, the EB schedule is built on a multi-slotframe structure, that is on a specific number of consecutive slotframe cycles. At the beginning of the slotframe there is a defined number of consecutive advertisement slots. As we will see next, both the length of the multi-slotframe structure as well as the number of advertisement slots depend on the number of advertisers and the desired EB rate. If we call *S* the number of slotframe cycles that compose the multi-slotframe structure, $A_{s}$ the number of advertisement slots in the slotframe, and C the number of available channels, then the total number of advertisement cells within the multi-slotframe structure is given by the following formula:

(10) $$A_{c}=S\times A_{s}\times C.$$

To distinguish the advertisement cells of the multi-slotframe structure, a unique index (i.e., a unique identifier) is defined for each of them. The cell indices are consecutive integer numbers starting from zero. Each advertiser transmits EBs in one of the advertisement cells, and utilizes its identifier (id) to find the index of this cell. Considering the node identifiers as integer numbers, each advertiser autonomously calculates the index ($cell_{idx}$) of its advertisement cell through the following formula:

(11) $$cell_{idx}=id\;\mathrm{mod}\;A_{c}.$$

To avoid the use of an advertisement cell by multiple nodes and, consequently, to avoid collisions, CFAS requires the following rule to be satisfied:

(12) $$id_{i}≢id_{j}\;\mathrm{mod}\;A_{c},$$

where $id_{i}$ and $id_{j}$ are the unique identifiers of any two nodes i,j. A necessary but not sufficient condition for this rule to be applied is the number of advertisement cells to be at least equal to the number of advertisers. When this condition is met, the rule can be simply satisfied if consecutive identifiers are given to the advertisers. This can also be easily satisfied if the advertisers get unique identifiers (not necessarily consecutive) within the range of 0 to $A_{c}-1$.

### 5.1. Advertisement Cell Indexing

Assigning unique indices to advertisement cells, which can be considered as a zero-based numbering of advertisement cells, can be done using various techniques. Herein, inspired by the literature, we consider the following two strategies:

1. Vertical indexing: starting from the first advertisement slot, the cells of each advertisement slot are numbered before the cells of the next advertisement slot. Within an advertisement slot the cells are numbered sequentially, from the cell of the lowest channel offset to the cell of the highest channel offset. When the advertisers have consecutive identifiers starting from 0, this method concentrates the EB transmissions into the smallest possible number of advertisement slots.
2. Horizontal indexing: beginning from the lowest channel offset, the cells of the same channel offset are numbered before the cells of the next channel offset. In the context of a channel offset the cells are numbered in time order; that is, the cell of the first advertisement slot is numbered first, the cell of the second advertisement slot is numbered second and so on. Assuming that the advertisers have consecutive identifiers starting from 0, this method distributes the advertisers as equally as possible among the advertisement slots.

**Figure 4:**
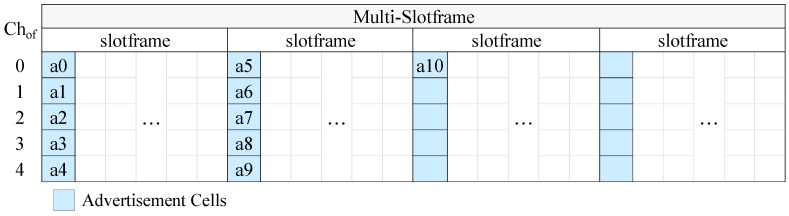
CFAS using vertical indexing in the case where there are 5 available channels, 11 advertisers (including the PAN coordinator), S=4 and $A_{s}=1$, while the identifiers of the advertisers are consecutive and start from 0.

**Figure 5:**
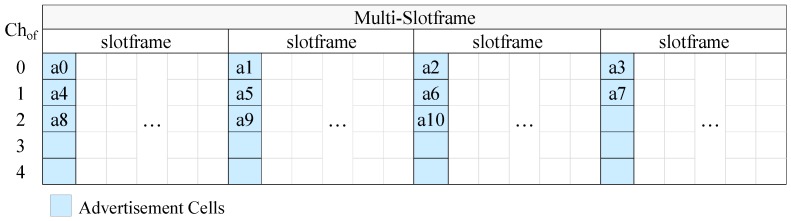
CFAS using horizontal indexing in the case where there are 5 available channels, 11 advertisers (including the PAN coordinator), S=4 and $A_{s}=1$, while the identifiers of the advertisers are consecutive and start from 0.

In Figure 4 we present an example of CFAS using vertical indexing, while in Figure 5 we present an example of CFAS using horizontal indexing. In these examples, we consider a network with 11 advertisers, including the PAN coordinator. The identifiers of the advertisers are consecutive and start from 0, the multi-slotframe structure consists of four slotframe cycles and the slotframe has one advertisement slot; that is, there are totally four advertisement slots in the multi-slotframe structure. For convenience, we consider only five available channels, and, consequently, only five channel offsets ($Ch_{o}f$). To avoid confusion, we note that a0 means advertiser with id 0, a1 advertiser with id 1, and so on.

**Figure 6:**
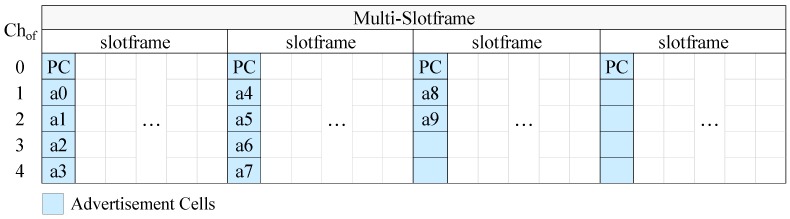
ECFAS using vertical indexing in the case where, except from the PAN Coordinator (PC), there are 10 other advertisers whose identifiers are consecutive and start from 0, the number of available channels is 5, S=4 and $A_{s}=1$.

**Figure 7:**
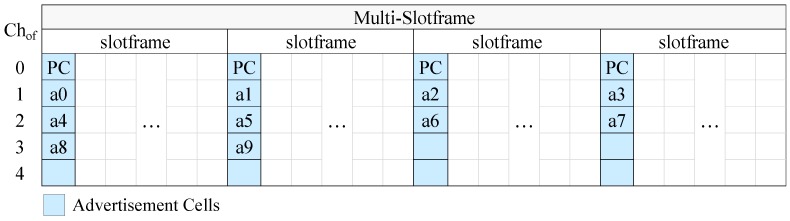
ECFAS using horizontal indexing in the case where, except from the PAN Coordinator (PC), there are 10 other advertisers whose identifiers are consecutive and start from 0, the number of available channels is 5, S=4 and $A_{s}=1$.

At this point, it should be noted that the PAN coordinator may be powered by the mains, in which case it can be constantly active and it can send EBs at a high rate in order to speed up the joining of its physical neighbors. For this case, we propose the “enhanced CFAS” (ECFAS) version, where the PAN coordinator sends EBs in all the advertisement slots, using the cells of channel offset 0, regardless of its identifier. These cells are not taken into account during the advertisement cell indexing; that is, the indexing starts from channel offset 1. If ECFAS is used instead of CFAS, then the examples of Figure 4 and Figure 5 are modified as shown in Figure 6 and Figure 7, respectively.

### 5.2. Advertisement Timeslot Partitioning

On the one hand, the assignment of different advertisement cells to advertisers solves the problem of collisions, but on the other hand it may lead to a high number of advertisement cells in the slotframe. Three are the factors that can lead to such a situation: (a) a high EB rate (i.e., a small multi-slotframe), (b) a large number of advertisers and (c) a limited number of channels. For example, in a network with 100 advertisers, 10 available channels, and a multi-slotframe consisting of only 1 slotframe, CFAS requires 10 advertisement slots; that is, with a typical slotframe of 101 slots, 10% of the slots are spent for EBs instead of data transmissions.

To tackle this problem we can use our previously proposed ATP technique [17]. ATP is a technique that aims at the optimal utilization of the available time of the advertisement slots in order to create more communication resources for EB transmissions. In this direction, ATP partitions each advertisement slot into smaller parts called subslots. A subslot is a compressed timeslot version that its size is as long as needed for the transmission of one EB.

**Figure 8:**
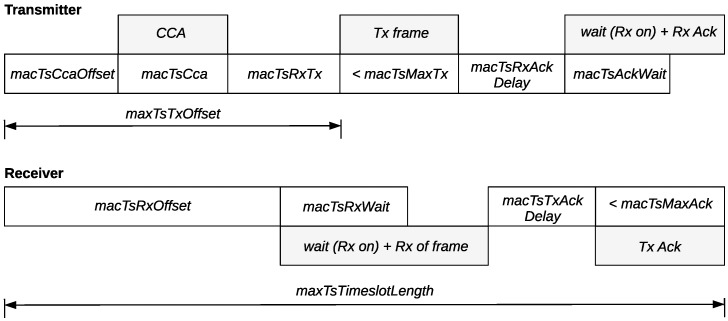
The IEEE802.15.4-TSCH timeslot template.

**Figure 9:**
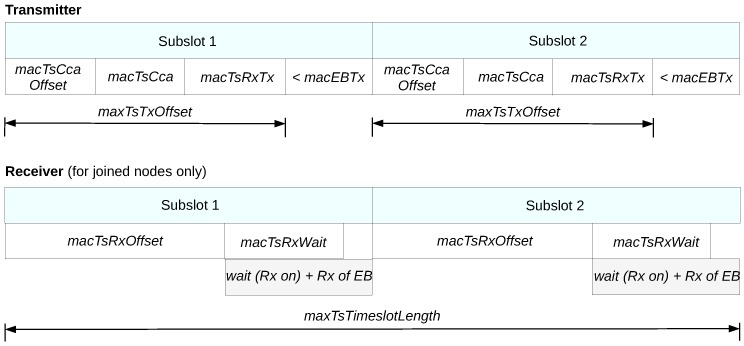
Advertisement timeslot structure with ATP using 2 subslots.

Figure 8 shows schematically the timeslot template of IEEE802.15.4-TSCH, while the attributes of the template are described in Table 1. Figure 9 presents the structure of an advertisement slot when ATP is used with 2 subslots. Apparently, the only differences between a standard timeslot and a subslot are the following. Firstly, since EBs are broadcast frames and are not acknowledged, the related time intervals are absent in a subslot. Secondly, the available transmission time in a subslot is not *macTsMaxTx*, but *macEBTx*, and it equals to the time required for the transmission of an EB. It must be noted here that the length of an EB and, consequently, its transmission time can be significantly reduced by using default identifiers in the Information Elements of the EB. The number of the subslots that can fit in an advertisement timeslot is given by the following formula:

(13) $$N_{subslots}=\left\lfloor \frac{macTsTimeslotLength}{macTsTxOffset+macEBTx}\right\rfloor$$

**Figure 10:**
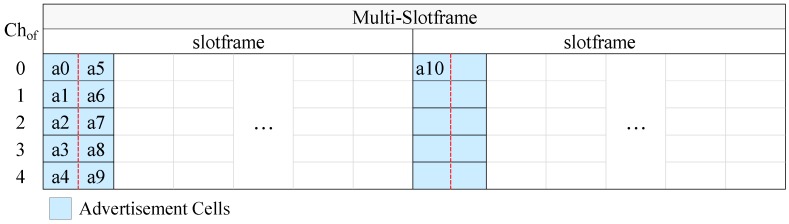
CFAS using vertical indexing and ATP in the case where there are 2 subslots per advertisement slot, 5 available channels, the identifiers of the advertisers are consecutive and start from 0, S=2 and $A_{s}=1$. The dashed lines separate the subslots of the advertisement slots.

Combining ATP and (E)CFAS, we can increase the number of advertisement cells without increasing the number of advertisement slots. It is worth noting that the use of ATP can be indicated by an extra Information Element in the EBs; this is supported by the standard and can be done with a negligible overhead on the EB size. In Figure 10 we present an example of CFAS combined with ATP, where each advertisement slot has two subslots, and, thus, there is a double number of available advertisement cells compared to the generic CFAS version.

In ATP, an advertiser transmitting in a sublot calculates the related channel using Equation (1), where ASN is that of the slot which the subslot belongs to. Therefore, the cells that are created by the sublots of an advertiserment slot in the same channel offset are mapped to the same channel. In order to generate different radio channels and boost the performance of (E)CFAS, we introduce the concept of the Serial Subslot Number (SSN). SSN is the serial number of the subslot within the slotframe that contains it, and is equal to the number of subslots elapsed since the start of the slotframe. Equation (14) treats the subslots in the same way as the generic TSCH channel generation function (i.e., Equation (1)) treats consecutive slots.

(14) PhysicalChannel=F{(ASN+ChannelOffset+SSN)%C}

## 6. Evaluation

To evaluate our proposed method we consider both the cases of: (a) a fixed joining node and (b) a mobile joining node. In both cases, we compare the above-mentioned versions of our proposed method to each other, as well as to ECV and ECH [5], which are the two most recently proposed non-centralized advertisement scheduling methods that are purely intended for IEEE802.15.4-TSCH. We also make a comparison with the well-known minimal 6TiSCH configuration [4]. Our simulations took place on an ad-hoc simulator (The code is available through the following link: https://github.com/akaralis/atjs), which we developed in Python. In order to provide realistic results, our simulator implements the general site path loss model recommended by ITU-R P.1238-9 [24] and takes into account the capture effect as described in the literature [25]. The general parameters of our simulations are presented in Table 2. To distinguish between the different versions of our method, we utilize the notations presented in Table 3. Regarding ATP, we assume that there are only 2 subslots per advertisement slot; that is, we take into account the smallest benefit that we can have by using ATP.

Our simulation results are finally enriched with a partially random cell assignment method that computes the advertisement cells based on the MAC addresses of the nodes. This method has been proposed in the literature [19] as a solution that can be easily implemented in real hardware in order to manage unicast data communications between neighboring nodes rather than a solution that completely eliminates collisions. It converts a node’s MAC address to an EUI64 address and, then, it applies a hash function to determine the slotframe offset as well as the channel offset. In our case, we use the result of the hash function to calculate the advertisement cell that an advertiser will use. The only difference with (E)CFAS is that in Equation (11)) we use the result of the hash function instead of the advertiser’s identifier. In correspondence with CFAS and ECFAS we consider two versions of this technique, namely the “MAC-based advertisement scheduling” (MAC-based AS) and the “enhanced MAC-based advertisement scheduling” (EMAC-based AS).

### 6.1. Study of a Fixed Joining Node

#### 6.1.1. Setup

In the case of a fixed joining node, we use as performance criterion the average joining time in relation to the number of the neighboring advertisers, which we consider as fixed nodes. We assume a range of 1 to 10 neighboring advertisers. For each method, in order to calculate the average joining time for a specific number of neighboring advertisers, we utilize samples from 1000 random topologies. In each of these topologies, each node starts its network operation at a random time within the first 100 s of the network initialization (i.e., the operation of the PAN coordinator). Moreover, each node gets a unique identifier that has been randomly selected from the integer interval $\left[0,k-1\right]$, where *k* is the number of available advertisement cells when (E)CFAS is used. In each of the random topologies, we initially wait for all the nodes to join the network and, then, we select one of them and perform 100 rejoining attempts. Each attempt finishes when the node receives a valid EB. In each method we collect a total of 10,000 samples for each examined number of neighboring advertisers. Due to the large sample, the confidence intervals are very small and it is difficult to depict them in the charts that we will present. However, we note that the confidence intervals (95%) are available in the dataset (the dataset is available through the following link: https://doi.org/10.6084/m9.figshare.7763528) that accompanies this paper.

#### 6.1.2. Comparison between Different Versions of CFAS

We start the evaluation of our method by comparing its various proposed versions. The goals of this comparison are to: (a) see if any of the particular advertisement cell indexing methods affects the performance, (b) confirm that ATP can be used without any compromise on the performance, and (c) calculate the speed up in the joining times of the PAN coordinator’s neighbors if we use ECFAS instead of CFAS.

**Figure 11:**
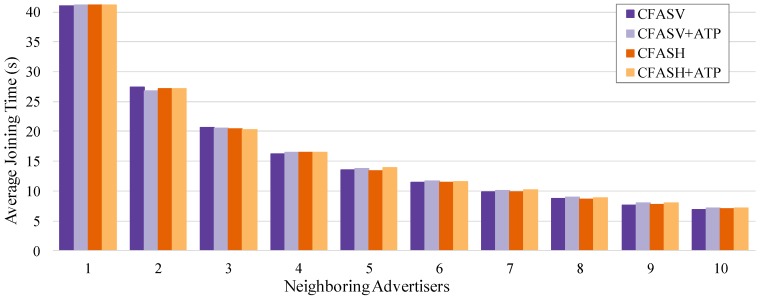
Comparison between CFASV and CFASH, with or without the use of ATP, in the case of a fixed joining node.

For the first two goals, we compare CFASV and CFASH, with and without ATP. The comparison is shown in Figure 11. It is obvious that without assuming specific identifiers for the neighboring advertisers, the advertisement cell indexing does not affect the performance. Furthermore, it is clear that ATP can be used without any compromise on performance.

**Figure 12:**
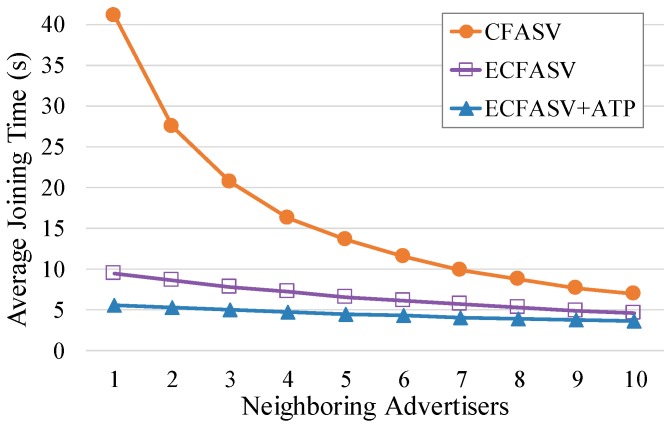
Comparison between CFASV and ECFASV when the joining node is a fixed neighbor of the PAN coordinator.

Since the advertisement cell indexing does not affect the performance, we can use CFASV as a representative of CFAS, and ECFASV as a representative of ECFAS. Then, we perform a comparison between CFASV and ECFASV to examine the benefits of using ECFAS when the joining node is a neighbor of the PAN coordinator. In this comparison, which is presented in Figure 12, we consider separately the case of using ECFASV with ATP, because in this case, the cells that the PAN coordinator transmits EBs increase, leading to an even higher EB rate for the PAN coordinator. As shown in Figure 12, ECFASV achieves much better average joining times in the neighborhood of the PAN coordinator compared to CFASV. Obviously, this is due to the increased EB rate of the PAN coordinator. In the best case, ECFASV achieves a 77% shorter average joining time without ATP and 86% when ATP is used.

#### 6.1.3. Comparison with Other Approaches

Next, we make a comparison with the minimal 6TiSCH configuration, ECV and ECH. For fairness reasons, we need to divide the compared methods in two categories: (a) in those that do not assume that the PAN coordinator has any power constraints, and (b) in the rest of the methods. The former category includes CFAS and the minimal 6TiSCH configuration, while the second includes ECFAS, ECV and ECH. Following this categorization, we will compare CFAS(V) with the minimal 6TiSCH configuration, and ECFAS(V) with ECV and ECH. In our comparisons we also include the simulation results of the MAC-based alternative of (E)CFAS.

**Figure 13:**
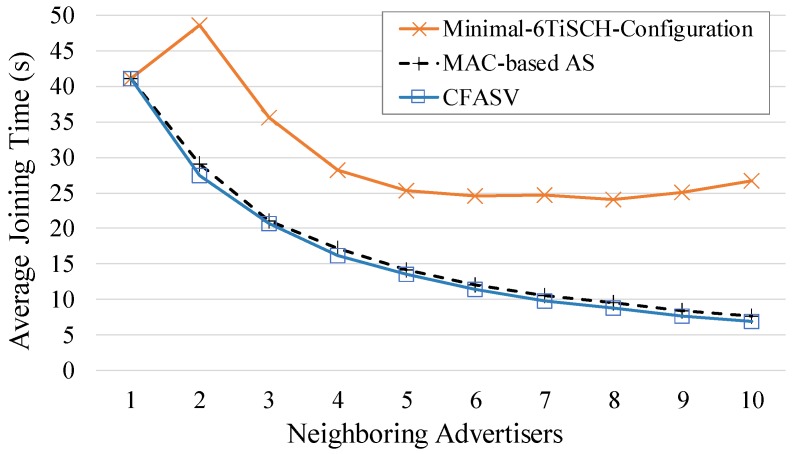
Comparison between CFASV, its MAC-based alternative and the minimal 6TiSCH configuration for a fixed joining node.

In Figure 13 we compare the average joining times of CFASV and of the minimal 6TiSCH configuration. We also present the average joining times of the MAC-based alternative of CFASV. It is straightforward to observe that CFASV is quite faster than the minimal 6TiSCH configuration. The performance difference reaches up to 74%. We must note here that based on the simulation parameters, each node sends an EB every five slotframes, and, hence, in the case of the minimal 6TiSCH configuration, there are a total of five advertisement cells (five distinct repetitions of the single advertisement cell of a slotframe). Therefore, according to the mathematical analysis shown in Figure 3, the minimal 6TiSCH configuration has the highest probability of full collision in the case of two neighboring advertisers, a fact that explains the high average joining time in this case. In general, it is obvious that the increased probability of a collision and, more specifically, of a full collision explains the poor performance of the minimal 6TiSCH configuration and its scalability weakness. Finally, our MAC-based approach performs slightly worse than CFASV since it does not completely eliminate the collisions.

**Figure 14:**
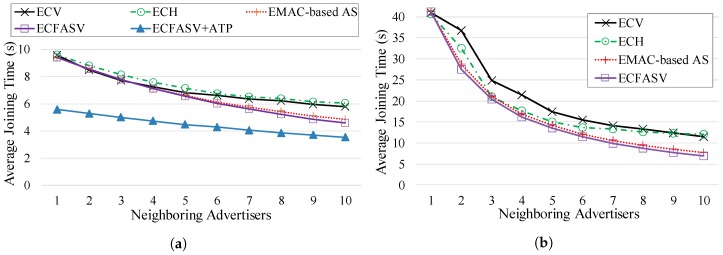
Comparison of ECFASV and its MAC-based alternative with ECV and ECH for a fixed joining node, when (**a**) the joining node is a neighbor of the PAN coordinator and (**b**) when not.

Regarding the comparison between ECFAS(V) and ECV/ECH, we consider two cases: (a) when the PAN coordinator is included in the neighboring advertisers and (b) when it is not included. Figure 14a,b respectively refer to these two cases. As we can observe, in both cases ECFAS is better than ECV and ECH. Apparently, this is due to the fully elimination of collisions that is achieved by ECFASV. In the first case, ECFASV achieves up to 42% shorter average joining time when ATP is used and up to 20% without ATP. In the second case, it achieves 42% shorter average joining time, regardless of the use of ATP, which in this case does not offer any improvement. Finally, the MAC-based alternative of ECFASV performs slightly worse than ECFASV since it does not completely eliminate the collisions.

### 6.2. Study of a Mobile Joining Node

#### 6.2.1. Setup

In the case of a mobile node, we assume that the network is enclosed in an area with dimensions of 100 × 100 m^2^. The fixed nodes of the network are the advertisers of the network that create a backbone around which the mobile node moves. The mobile node moves according to the random waypoint model [28], with a speed range of 0.1–5 m/s and zero pause times at the waypoints. The reception of an EB (or frame) of maximum size lasts for around 4256 μs, considering negligible the propagation delay. This time is very small and allows the reception of an EB even if a mobile node moves at the highest speed that we considered in the simulations. At this point, it is worth noting that we can consider that the mobile node, during its movement, collects data (e.g., temperature) and periodically or after a specific event defined by the network requirements, tries to join the network in order to forward the data to the network coordinator. Since the neighboring advertisers of the mobile node change due to mobility, we calculate the average joining time in relation to the density of advertisers in the network area. The sampling method that we use is similar to the case of a fixed joining node; that is, we collect samples from different random topologies by making rejoining attempts of the mobile node at random times. For each examined method, we consider 10 to 150 advertisers with an increment of 20.

Similar to the case of a fixed joining node, we compare CFAS(V) with the minimal 6TiSCH configuration, and ECFAS(V) with ECV and ECH. In each case, we also evaluate our MAC-based alternative of our proposed method. We note that, for purely formal reasons, we have confirmed through simulations that even in the case of a mobile joining node the advertisement cell indexing does not affect the performance.

#### 6.2.2. Comparison with the Literature

**Figure 15:**
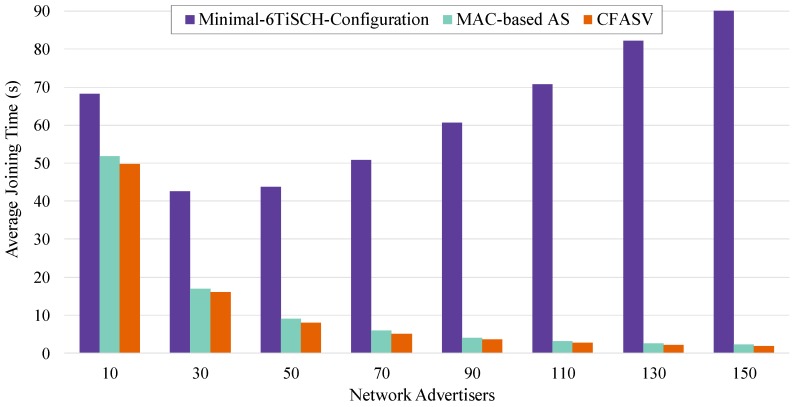
Comparison of CFASV and its MAC-based alternative with the minimal 6TiSCH configuration for a mobile joining node.

In Figure 15 we compare the average joining times of CFASV and its MAC-based alternative with those of the minimal 6TiSCH configuration. Compared to the minimal 6TiSCH configuration, CFASV performs much better and achieves multiple times lower average joining times. Again, as in the case of the fixed joining node, we observe that the minimal 6TiSCH configuration has very poor performance and it is not scalable. The high probability of a collision and, mainly, of a full collision is the cause of this problem. Indeed, by using Equation (9) we can see that the full collision probability in our simulation scenario of the minimal 6TiSCH configuration is constantly increasing for neighboring advertisers greater than five and is almost 100% when the neighboring advertisers are more than 30. It is obvious that the use of a single advertisement cell per slotframe results in a poor performance due to the collisions. Therefore, there is a need for a mechanism that dynamically adapts the EB rate in a way that minimizes the collisions without degrading the joining time. As we mentioned in Section 3, such a mechanism has been proposed in Ref. [9] and its evaluation showed that it actually improves the performance of the minimal 6TiSCH configuration, but nevertheless it does not solve its scalability problem. Moreover, it should be noted that this mechanism has been defined in the context of a fully-meshed network, and, thus, there is a need for a general solution. Finally, our MAC-based approach performs slightly worse since it does not completely eliminate the collisions.

**Figure 16:**
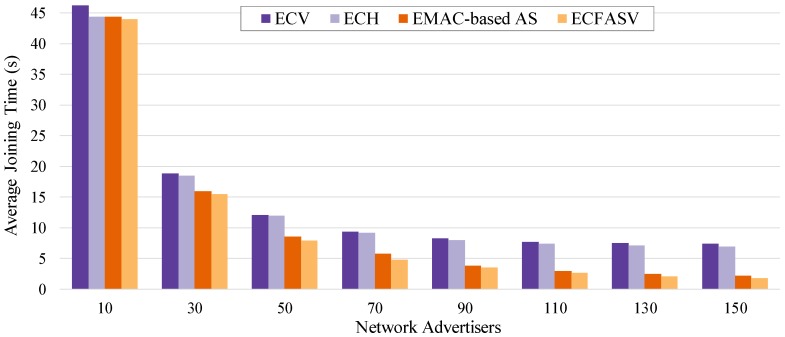
Comparison of ECFASV and its MAC-based alternative with ECV and ECH for a mobile joining node.

Subsequently, in Figure 16, we compare ECFASV to ECV and ECH. For presentation purposes, we also include results of the MAC-based approach. It is straightforward to see that the collision policy of ECFASV affects the performance positively; ECFASV achieves better results in all the cases and the performance difference increases as the number of advertisers increases. Apparently, in contrast to ECV and ECH, the collision-free nature of ECFASV allows the full exploitation of the aggregated EB rate of advertisers in order to minimize the joining time. In the best case, ECFASV exhibits a 75% better average joining time than both ECV and ECH. Finally, when the cells are assigned based on the MAC address of the nodes, the randomness is limited, resulting in a smaller number of collisions without, however, completely eliminating them.

### 6.3. Study of the Energy Consumption

In this subsection we measure the total energy consumption of the nodes until all the nodes connect to the network. To do so we take into account a realistic energy consumption model similar to the one considered in 6TiSCH Simulator [29]. In this study, 10% of the nodes are mobile.

**Figure 17:**
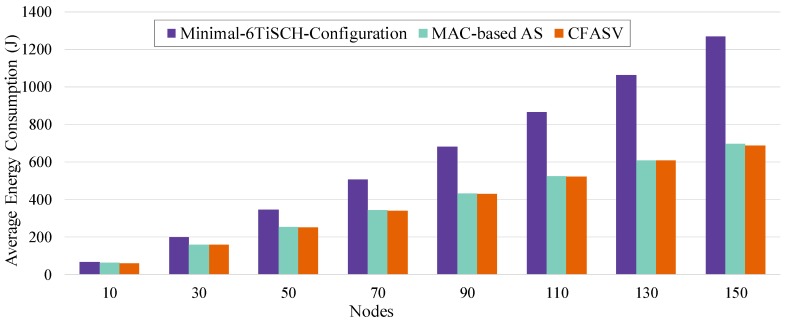
Total energy consumption until all the nodes connect to the network for a scenario where the PAN coordinator has limited power resources.

**Figure 18:**
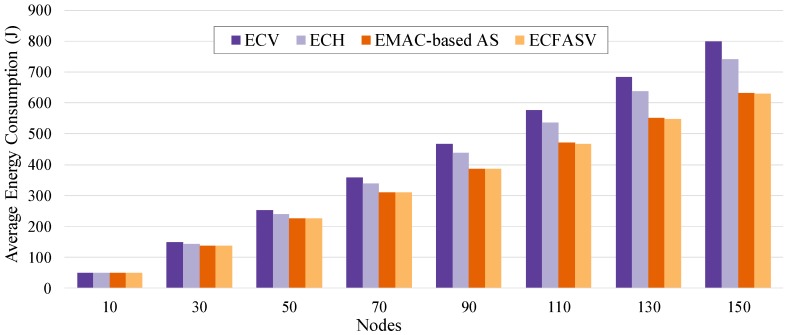
Total energy consumption until all the nodes connect to the network for a scenario where the PAN coordinator has unlimited power resources.

Figure 17 illustrates the results of a scenario where the PAN coordinator has limited power resources. We can observe that CFASV achieves much less consumption compared to minimal 6TiSCH configuration. When CFAS is combined with the MAC-based AS technique, the performance is slightly lower due to the additional active time of the nodes until they finally connect to the network. This additional time is higher in MAC-based AS because of the non-zero probability of collisions. In Figure 18, we compare ECFASV to ECV and ECH as well as to the Enhanced version of MAC-based AS. The results show energy consumption gains that vary from 2% to 21%. This is a clear evidence that the reduced joining time leads to a reduced energy consumption as well.

## 7. Conclusions and Future Work

In this paper we dealt with the problem of EB scheduling in IEEE802.15.4-TSCH networks and, more precisely, with the analysis and addressing of the issue of collisions, which adversely affects the performance, leading to long joining times and, thus, to increased power consumption. In this context, we showed that random-based EB scheduling methods still lead to a high probability of collisions and, especially, of full collisions. To address this issue, we proposed CFAS, a novel autonomous EB scheduling approach, which in contrast to the other non-centralized approaches in the literature, eliminates the collisions. Our simulation experiments showed that our approach achieves a significant improvement in terms of average joining time without increasing the energy consumption.

In the future, we are planning to implement CFAS in a real hardware platform. We will also investigate how CFAS can be combined with other types of traffic, such as with routing and data packets. It is important to see the synergies between routing and advertisement activities, while not compromising the data scheduling performance. We will need to observe whether routing data can fit together with advertisement data within a single slot using ATP in order to decrease the number of cells used for control messages and leave more available slots for data transmissions. Another interesting topic to explore is the adoption of a dynamic advertisement strategy in order to adapt the EB rate according to the data traffic. Possible trade offs between joining times and data latency will need to be solved.

## Figures and Tables

**Table 1:** The attributes of the IEEE802.15.4-TSCH timeslot template.

Attribute	Description	Default Value (2.4 Ghz Band)
macTsCcaOffset	The time between the start of the timeslot and the beginning of the Clear Channel Assessment (CCA) operation.	1800 μs
macTsCca	The duration of the CCA operation.	128 μs
macTsRxTx	Time available for transition from receive mode (after the CCA operation) to transmission mode	192 μs
macTsTxOffset	The time between the start of the timeslot and the start of the frame transmission.	2120 μs
macTsMaxTx	Transmission time to send the maximum length frame.	4256 μs
macTsRxAckDelay	The time between the end of the frame transmission and the time when the transmitter starts listening for the acknowledgement (if an acknowledgment is required).	800 μs
macTsAckWait	The minimum waiting time for the start of an acknowledgment.	400 μs
macTsRxOffset	The time between the start of the timeslot and the time when the receiver starts to listen.	1020 μs
macTsRxWait	The time to wait for the start of the frame.	2200 μs
macTsTxAckDelay	The time between the end of the frame and the start of the acknowledgment (if an acknowledgment is required).	1000 μs
macTsMaxAck	Transmission time to send an acknowledgment.	2400 μs
macTsTimeslotLength	The total length of the timeslot including any unused time after the transmission of a frame and its acknowledgment.	10,000 μs

**Table 2:** General simulations parameters.

Parameter	Description	Selected Value
Slotframe Length	The length of the slotframe	101 slots
Multi-slotframe Length	The length of the multi-slotframe structure. It is identical to the enhanced beacon interval (i.e., the interval between two consecutive EB transmissions of an advertiser).	5 slotframes
Advertisement slots (or cells)	The number of advertisement slots (or cells) in the slotframe	All the compared methods except the Minimal 6TiSCH configuration use one advertisement slot, while the minimal 6TiSCH configuration uses the recommended one shared cell [4].
Number of Channels	The number of available channels	16 (2.4 Ghz band)
Timeslot Template	The values of timeslot attributes	The default timeslot template of the 2.4 Ghz band (as defined by the standard)
Scan Duration	The time that a joining node scans a channel to find an EB	2 × Multi-sloframe Length
Channel Switch Delay	The time elapsed when changing to a new channel, including any required settling time. According to the standard, this time shall be less than or equal to 500 μs [3].	200 μs (approximately as much as in a Chipcon CC2420 radio chip [26])
Channels Scan Sequence	The order in which a joining node scans the channels to find an EB	As defined by the standard (i.e., channels are scanned in order from the lowest channel number to the highest)
Path Loss Model	The model that describes the signal attenuation between a transmit and a receive antenna.	The general site model recommended by ITU-R P.1238-9 [24], including slow fading
Capture Effect Threshold	Conforming to the literature, our simulator requires the strongest frame to arrive either first or within the synchronization header of the first weaker frame [25].	3 dBm (according to the literature [25])
Power Consumption Model	The power consumption model of nodes. We use the power consumption details of Zolertia RE-Mote Revision B. [27]	Transmission: 24 mA Reception: 20 mA Listening: 20 mA Sleep mode: 1.3 μA

**Table 3:** Notations and descriptions for the different versions of collision-free advertisement scheduling (CFAS).

Notation	Meaning	Description
CFAS	Collision-free advertisement scheduling	This is the main proposed algorithm of the paper described in Section 5.
CFASV	CFAS with vertical cell indexing	This indicates the type of cell indexing that can be used for CFAS. Vertical or Horizontal cell indexing can be used as explained in Section 5.1.
CFASH	CFAS with horizontal cell indexing	
ECFASV	Enhanced CFAS with vertical cell indexing	In the Enhanced version of CFAS, the PAN coordinator is allowed to use all the available advertisement cells of channel offset 0. This version of CFAS can be used when the PAN coordinator has unlimited power resources.
ECFASH	Enhanced CFAS with horizontal cell indexing	
(E) CFASV + ATP	(E)CFASV together with ATP	All the versions of CFAS can be further enhanced with the “advertisement timeslot partitioning” technique, where multiple EBs can be sent in a single timeslot.
(E) CFASH + ATP	(E)CFASH together with ATP	
